# Big Talks for Little People: A Pilot Study of a Classroom Based Mental Health Program

**DOI:** 10.1002/hpja.70014

**Published:** 2025-02-21

**Authors:** Phillip Slee, Shane Pill, Deborah Agnew

**Affiliations:** ^1^ Flinders University Adelaide Australia

**Keywords:** anxiety, bullying, emotional expression, mental health, social emotional learning

## Abstract

**Background:**

Schools are important settings for the promotion, implementation and education of mental health and well‐being. The present study piloted and evaluated a classroom based mental health and well‐being programme for use in primary schools.

**Methods:**

A mixed‐method quasi‐hybrid design methodology was utilised. An online survey was completed by students from five schools pretest (*n* = 173) and three schools matched post‐test (*n* = 68) with semi‐structured interviews with teachers (*n* = 4) and a focus group of students (*n* = 18) conducted at the completion of the programme. Student questionnaire data was gathered including the use of three standardised and internationally used measures of well‐being.

**Results:**

Students at post‐test self‐reported significant improvements in positive emotional state (*p* < 0.05, moderate effect size), recognising (*p* < 0.001, large effect size) and expressing emotions (*p* < 0.001, moderate effect size) and reductions in anxiety (*p* < 0.001, moderate effect size).

**Conclusions:**

The findings suggest the programme was effective in relation to promoting aspects of student well‐being, emotional development and in reducing elements of anxiety. Shortcomings in the design including a lack of a control group must lead to caution in interpreting the outcomes.

**So What:**

Further research with a larger student population which addresses the identified shortcomings of the present pilot study appears warranted.

## 1 Introduction

Mental health issues have been recognised as the most pressing problem for children in developed countries [1]. In Australia, the site of this study, approximately half of children's diagnosed mental health issues onset before 14 years of age [2]. Mental health concerns are common among primary school aged children and left to persist, can progress to long‐term problems with anxiety, depression, hyperactivity and aggression, reduced capacity to engage with schooling and issues with forming and maintaining positive peer relationships [3]. The aim of this study was to evaluate an online primary school classroom‐based, teacher‐delivered mental health education programme. As noted by Dix et al. [4, 5], in the Australian context there is relatively limited research evidence for the efficacy and effectiveness of many well‐being and health education programmes.

The focus was on early intervention and prevention for all children using social emotion learning (SEL) principles for which there is sound evidence concerning their effectiveness [6, 7]. SEL programmes are used by many schools worldwide with evidence they lead to significant reductions in students' emotional distress, behaviour problems and mental health concerns [8, 9]. Improvements in social and emotional competencies, classroom behaviour, peer relationships and engagement and academic success are also noted [10]. Research also found that students' school attendance and motivation to learn improve [8]. These positive outcomes have been shown to be sustained on average for between 1 and 3 years [9].

SEL programmes targeted at schools generally have a broad focus on promoting healthy outcomes, including academic attainment, family relationships and social ability [8]. Programmes may also target reducing disruptive behaviours, including physical and relational aggression, problem‐solving, communication skills and empathy development [11, 12]. Programmes focused specifically on bullying prevention have concentrated on assertiveness, being an active bystander and keeping safe.

Students who report being bullied often state they feel unsafe at school, which is concerning given feeling safe is a protective factor for bullying [13]. We note here that for the behaviour to be defined as bullying, it must be repetitive, and there must be a power imbalance whereby it is difficult for the victim to stand up for themself. When teachers fail to address bullying, this antisocial behaviour tends to continue and sometimes escalate. When teachers attempt to address bullying by coercive and/or negative behaviour management strategies, these behaviours can be adopted by students in their peer interactions. When the teacher uses positive behaviour management strategies that demonstrate positive social behaviours, and direct students to these behaviours, it has been found that these values may also be adopted by students in their peer interactions [13]. Bullying and related forms of ‘othering’ in both implicit and explicit ways, needs to be educatively addressed by teachers to foster the SEL competency of ‘inclusion’ [14]. For example, DeLay et al. [15] found that students participating in a relationship building programme for promoting acceptance and inclusion developed greater diversity of friendships selection. As such, positive social behaviours are important for establishing harmonious peer relationships which, in turn, is associated with a reduction in bullying [16].

Taylor et al.'s [9] evaluation of 82 school based SEL interventions, found positive, long‐terms effects of SEL interventions with children and youth. The SEL interventions were found to improve student–peer relationships and academic achievement and to decrease in school behaviour problems. The same authors emphasised the benefits of evidence‐based, and teacher‐implemented SEL interventions. The benefits include significant effects on social skills and effects on academic outcomes, and problem behaviour [4, 10]. The review conducted by Dix et al. [4] concluded that well‐being programmes that include the development of teacher content knowledge (capacity) are more effective than programmes delivered by external professionals. This suggests teacher professional development focussed on improving their SEL content knowledge is essential for such programmes to influence student well‐being outcomes. The ‘Big Talks for Little People’ programme has addressed this shortcoming by including an online component of teacher professional development which must be completed before commencing delivering the programme. Aware teachers have the ability to notice student mental health issues [17], and there is a growing expectation on teachers to understand behaviour associated with common mental health concerns in young people. Not surprisingly, therefore, teachers are looking for training and development in mental health education [18, 19]. The Big Talks for Little People programme aims to address the identified need for early intervention in primary school [3].

## 2 Method

A mixed‐method quasi‐hybrid design methodology was utilised for this project [20, 21]. This design was considered to fulfil the pragmatic requirements for conducting programme evaluation research in the naturalistic setting of the intervention which saw classroom teachers delivering a mental health education programme to primary (elementary) school students [22]. Researchers [23] have argued that this approach has advantages in implementation science research. This paper focusses on the quantitative data gained from the project. The project received approval from the authors University Human Research Ethics Committee, Catholic Education South Australia Research Ethics Committee, the Department for Education Ethics Committee and the Australian Association for Research in Education National Application Form.

## 3 Participants

Five schools were recruited from a pool of eight schools who had heard about the programme and approached Author 1 for more information. In choosing the schools, maximum variation sampling [24] was employed based on the 7‐point Index of disadvantage used in South Australia whereby Category 1 schools indicates the most socio‐economically disadvantaged communities and Category 7 indicates the least disadvantaged. The spread of the five schools across the Index varied from 2 to 7.

The programme is premised on social systems theory [13] and consisted of a mental health education curriculum that included digital animations, multimedia activities and SEL teacher information sheets (e.g., bullying, trauma informed teaching). The content for the programme was informed by an extensive literature review, interviews with nine well‐being counsellors, teachers and child psychiatrists with expertise in the mental health field and curriculum specialists. The mental health literacy development was designed for the specific primary school age. The programme provided teachers with:

- Six 40‐min lessons aligned with the Australian Curriculum General Capabilities [25, 26];
- Short digital animations for each lesson (five to six animations) to provide stimulus for SEL discussions with students; and
- Six teacher information sheets on SEL topics.

A key element helping ensure implementation quality noted by Dix et al. [4] review is that the teachers delivering programmes receive professional development on the programme and its content. In this regard, all the teachers who were involved in delivering the Big Talks for Little People programme received professional development (1–2 h duration) that was delivered by two experienced researchers before the teachers implemented the programme [27]. As part of the professional development, teachers were introduced to and registered for an online platform housing the programme and its related content. https://www.bigtalksforlittlepeople.com.au/.

A first draft of the programme was tested using a convenience sample of three teachers and four young people to ensure that the content ran seamlessly on the digital smartboards of the classrooms and to test the links to the online questionnaire.

The evaluation of the programme pilot involved quantitative data gathered: (i) pretest from 173 students and (ii) matched pre and post test data from 68 students (Table 1). Note there was missing data in the demographics.

**TABLE 1:** Demographic details of students completing the pre and matched post‐test survey.

Pretest (*n* = 173)		Post‐test (*n* = 68)
Age (years)	8–11	9–12
Year level	4–6	4–6
Gender (%)	Male 35	Male 35
	Female 64	Female 62

The online survey completed by students generated data that was collected pre‐ and post‐test for a number of variables relevant to well‐being and mental health, and these are listed in Table 2.

**TABLE 2:** Key variables and scale details analysed for the pilot study.

Item	Variable
Face most like me at school	Happiness at school (5 point)
Happy about interpersonal skills	Personal competence (5 point)
Happy about schoolwork	Academic competence (5 point)
Free to be myself at school	Self‐expression (5 point)
Stirling well‐being scale (StirCWB)	Total well‐being (5 point)
Emotion Expression Scale Children (EESC)	Poor emotional awareness (5 point)
The Social Anxiety Scale for Children (SASC‐R)	Social anxiety (5 point)
Bullied at school (frequency)	Self‐reported victimisation (7 point)
Quality of peer relationships	Peer relationships (5 point)
Pride in belonging to school	School connectedness (10 point)
Satisfied with self	Self‐concept (10 point)
A person of worth	Self‐esteem (10 point)

*The Stirling Well‐being Scale for Children (StirCWB Scale)* was developed for students aged 8–15 years [28]. Research piloted with children demonstrated that the StirCWB Scale to have good theoretical grounding, and was able to be understood, and perceived by children of this age group to be measuring their well‐being [28]. The StirCWB Scale consists of 12 items that assesses emotional and psychological well‐being, positive emotional state (PES) and positive outlook (POL). Measurement is by a 5‐point Likert type scale with the range being from 1 (*never*) to 5 (*all of the time*) as a measurement of how well each item on the scale describes their well‐being during the previous 3–4 weeks.

*The Emotion Expression Scale for Children (EESC)* is a self‐report scale for primary school aged students. It examines two elements of deficient emotion expression, namely, (i) lack of emotion awareness and (ii) lack of motivation to express negative emotion. Difficulties with recognising emotion and expressing emotion have been linked to poor mental health and well‐being [29].

Saarni [30], found emotion awareness and expression to be components of emotional competence that contribute to student development of ‘a sense of subjective well‐being and adaptive resilience in face of future stressful circumstances’ (p. 10). Emotion awareness enables the ability to be aware of and address one's own emotions [31]. This is a key ability to be able to cope with an emotional experience [32]. The EESC scale uses a 5‐point Likert‐type scale ranging from 1 (*not at all true*) to 5 (*extremely true*) to indicate students' experience with expressive difficulties. As reported by Penza‐Clyve and Zeman [29] the scale is normally distributed with higher scores indicating low emotion awareness and more reluctance to express emotion.

*The Social Anxiety Scale for Children–Revised (SASC–R)* [33, 34] is a 22‐item scale for the purpose of capturing the different between subjective and behavioural aspects of social anxiety. The SASC‐R is rated on a 5‐point Likert scale from 1 (*not at all*) to 5 (*all the time*). However, in the present study a 7‐point Likert scale was used to improve the discrimination as suggested by Taherdoost [35]. The SASC–R contains three factors: (1) Fear of Negative Evaluation (FNE); (2) Social Avoidance and Distress Specific to New Situations (SAD–New) and (3) General Social Avoidance and Distress (SAD–G) [36].

## 4 Results

The data analysed and presented in Table 3 involved the students who completed the online questionnaire pretest (*n* = 173) and 88 students who completed the post‐test. However, not all of the post‐test students (*n* = 88) could be matched because they were absent on the day. This resulted in a matched pre and post‐test sample of 68 students from three schools.

**TABLE 3:** Details of results for students (unmatched) completing the pre and post‐test survey (*n* = 173).

Item	Variable	Pretest frequency (*N* = 173)	Post‐test (matched) frequency (*N* = 68)	Change
Face most like me at school	Happiness at school	6.3% unhappy (5–7)	2.7%	Improved
How many friends at school	Friendship network	6.9% (1 or no friends)	5.3%	Improved
Happy about skills	Personal competence	2.8% report lack of competence	0%	Improved
Happy about schoolwork	Academic competence	8.1% unhappy about school studies	10.7%	Declined
Total well‐being scale	Total well‐being (high score = improved well‐being)	*X* = 43.50	*X* = 44.01	Improved
Total emotion expression children (EESC)	Total EESC (low score = improved emotion awareness)	*X* = 45.19	*X* = 42.96	Improved
Fear of Negative Evaluation	Worrying about evaluations by peers (high score indicates more worried)	*X* = 18.63	*X* = 18.50	Improved
Bullied at school	Self‐reported victimisation (high score indicates greater victimisation)	19.4%	21.3%	Increased
Get along with other students	Peer relationships (high score indicates poorer peer relations)	12.7% do not get along with peers	12.3%	Improved
Satisfied with self	Self‐concept (high score indicates poorer self‐concept)	10.6% dis‐satisfied with self	9.3%	Improved
A person of worth	Self‐esteem (high score indicates poorer sense of worth)	10.5% report low self‐esteem	6.7%	Improved

In relation to this unmatched sample of 173 students from five pilot schools, the trends overall in the students' ratings pre‐ and post‐test indicated small improvements in students' reported school relationships, well‐being, emotional expression, self‐concept and self‐esteem. The level of self‐reported victimisation increased slightly and happiness with academic competence declined. The findings regarding the unmatched sample of students from the 5 schools are presented to provide the reader with a broad understanding of the trends relating to a number of variables that students reported on in their online questionnaires.

Overall, utilising the self‐reported data for the 173 unmatched student's responses from five schools to a range of measures it can be seen that the overall trend was for improvement across the 6‐week programme. The general picture emerging suggests improvements in student happiness, connection to school, personal competence and well‐being. There were reductions in ratings for academic competence and a reported increase in being bullied. However, a more accurate assessment of the effectiveness of the intervention follows in reporting data from the matched sample of 68 students pre‐ and post‐test.

Following Liddle and Carter's [37] protocol, student responses to the StirCWB Scale were used to calculate a total well‐being score for each respondent. The StirCWB individual scores ranged from 15 to 60 with the mean 43.27 (standard deviation [SD] = 9.09). This is slightly lower than the mean StirCWB score in the Liddle and Carter study, which was 43.5 (SD = 6.7). The mean score from our study was within an acceptable one SD of the mean in Liddle and Carter's study. In our study, a small number (8.4%) of students had scores less than 30. Liddle and Carter [37] suggest scores less than 30 indicate poor mental health (languishing vs. flourishing). This proportion of scores less than 30 in our study is comparable with the proportion of students found to be languishing (6.1%) by Keyes [38].

Confirmatory factor analysis (CFA) determined the presence of the two sub‐components of the StirCWB Scale. The two‐component solution explained 61.49% of the variance with Component 1 contributing 53.38% and Component 2 contributing 8.11%. An oblim rotation was performed and the rotated solution showed the presence of a simple structure with both components showing strong loadings on all variables.

Paired samples *t*‐tests were appropriate given that the data being analysed met the various criteria for parametric tests and were conducted to evaluate the impact of the intervention on Total Well‐being, POL and PES.

In relation to Total Well‐being there was no statistically significant difference pre and post‐test (*p* < 0.09) for the intervention although the mean scores for pretest (= 44.33) and post‐test (= 46.23) indicated that the higher scores at post‐test showed an improvement in student well‐being. Analysis by gender showed no significant difference pre and post‐test although the mean scores for both males (pretest = 43.38 and post‐test = 43.95) and females (pretest = 44.88 and post‐test = 47.55) showed improvements in total well‐being.

In relation to the component of POL paired sample *t*‐tests showed no significant difference associated with the intervention, although POL improved from pretest (= 22.75) to post‐test (= 23.27). In relation to PES, there was a significant improvement from pretest to post‐test (= 21.52, SD = 5.10) to post‐test (= 22.85, SD = 4.24, *p* < 0.05). The eta squared statistic 0.06 indicated a moderate effect size. Overall, these findings provide evidence that a 6‐week programme is long enough to effect positive change in relation to students' hedonic outlook. That is, overall pleasure, enjoyment and satisfaction and the absence of distress.

CFA determined the presence of the two sub‐components of the EESC Scale. The two‐component solution explained 43.56% of the variance with Component 1 contributing 34.76% and Component 2 contributing 8.80%. An oblim rotation was performed and the rotated solution showed the presence of a simple structure with both components showing strong loadings on most variables. Paired samples *t*‐tests were conducted to evaluate the impact of the intervention on the two components of the EESC Scale, namely, (i) Expressive Reluctance Factor and (ii) Poor Awareness Factor. In relation to the Expressive Reluctance factor, there was a significant improvement from pretest to post‐test (= 24.00, SD = 5.98) to post‐test (= 20.66, SD = 5.83, *p* < 0.001). The *η* ^2^ statistic 0.23 indicated a large effect size. In relation to the Poor Awareness factor, there was a significant improvement from pretest to post‐test (= 22.62, SD = 6.70) to post‐test (= 20.66, SD = 5.83, *p* < 0.001). The *η* ^2^ statistic 0.08 indicated a moderate effect size. Overall, these findings provide some evidence that a 6‐week programme can effect positive change in relation to students' ability to express emotion and their awareness of emotions. This would be consistent with the strong focus of the lesson content and animations on identifying feelings and emotions and managing appropriate ways to express them.

Four items comprising the FNE component were analysed. Scores ranged from 4 to 28 using an adapted 7‐point Likert Scale (intended to enable greater differentiation than a 5‐point scale) with a mean score of 14.51 and SD of 7.70. A paired sample *t*‐test showed no significant difference for the total matched sample (*n* = 68). Analysis was then undertaken of the data using a cut‐off point one SD above and below the mean to identify two groups—(i) students with a low score on the FNE scale and students with a high score on the FNE scale. Paired sample *t*‐tests showed no significant difference for students with a low score on FNE. However, students with a high score on FNE at pretest (= 23.91, SD 2.84) scored significantly lower at post‐test (= 18.50, SD 6.19) indicating less concern with other peers' negative evaluations. The eta squared statistic 0.39 indicated a moderate effect size. This would suggest that the intervention possibly results in less FNEs by peers for those who score highly on this factor. One interpretation of this finding is that the anxiety of being negatively evaluated, for example, peers are making fun of them, reduces across the intervention period.

Analysis was undertaken of the pretest data (*N* = 173) to better understand the strength and direction of relationships between school bullying and (i) peer relationships, (ii) happiness at school, (iii) self‐concept and (iv) self‐esteem. The relationship between self‐reported bullying by others and happiness at school was investigated using Pearson's product–moment correlation coefficient. Preliminary analyses were undertaken to ensure no violation of the assumption of normality, linearity and homoscedasticity. A strong negative correlation between the two variables, *r* = −0.331, *p* < 0.01 such that high levels of self‐reported bullying were significantly associated with unhappiness at school were found. The relationship between self‐reported bullying by others and peer friendships at school was investigated using Pearson's product–moment correlation coefficient. Preliminary analyses were performed to ensure no violation of the assumption of normality, linearity and homoscedasticity. There was a strong negative correlation between the two variables, *r* = −0.431, *p* < 0.01, such that low levels of self‐reported bullying were significantly associated with positive peer relationships.

The relationship between self‐reported bullying by others and positive self‐concept at school was investigated using Pearson's product–moment correlation coefficient. Analyses were undertaken to ensure no violation of the assumption of normality, linearity and homoscedasticity. A strong negative correlation between the two variables, *r* = −0.222 *p* < 0.01, such that high levels of self‐reported bullying were significantly associated with negative self‐concept was found. The relationship between self‐reported bullying by others and self‐esteem at school was investigated using Pearson's product–moment correlation coefficient. Preliminary analyses were conducted to ensure no violation of the assumption of normality, linearity and homoscedasticity. A strong negative correlation between the two variables, *r* = −0.406, *p* < 0.01, such that high levels of self‐reported bullying were significantly associated with low self‐esteem was found.

Finally, using the matched pairs sample (*n* = 68) it was found that the level of student self‐reported bullying was reduced by 25% across the intervention period. This finding is not inconsistent with the strong focus of the lesson content and animations on the importance of building positive peer relations and friendship making skills.

## 5 Discussion

Overall, evidence was found that the Big Talks for little People programme positively impacted important aspects of primary school children's well‐being and mental health. In relation to student well‐being, the present findings were well aligned with the international research. Males were more likely than females to show signs of positive well‐being, with more males than females showing a POL and a PES. This type of gender difference is not a uncommon. For example, Kuter and Deom [39] reported that girls' daily feelings of sadness or hopelessness were more likely than those reported by males. A more negative perspective by girls is generally reported in the literature and appears to widen with age. Possible explanations could include the negative impact of social media and/or may be associated with transitioning through puberty and negotiating pressures associated with body image and appearance [40], It has also been argued that it is not necessarily that males experience less feelings of distress than females, rather, mental distress and poor well‐being are less likely to be disclosed by males [41]. Research suggests that social norms regarding what it means to be masculine may result in an underreporting of symptoms by males [41].

Considering the effectiveness of the intervention, no significant difference between student pre and post‐test for total well‐being was found, although the mean scores indicated that well‐being was higher at post‐test. In relation to the factor of ‘Positive Emotional State’ (hedonic) there was a significant improvement of moderate effect size across the 6‐week intervention. This would suggest that the lessons and animations were effective in promoting a stronger sense of pleasure, enjoyment and satisfaction. Some support for this is found in an examination of other variables assessed in the online questionnaire where post‐test students reported greater happiness at school, a stronger friendship network and less bullying.

In terms of the analysis regarding the EESC, there were significant improvements in students' willingness and ability to express their feeling and an improved awareness of their feelings and emotions across the 6‐week intervention. To the authors best knowledge this is one of the few evaluations of an EESC as an intervention.

The lesson content and the animations of the programme focussed strongly on helping primary school students recognise and identify their emotions and feelings. One interpretation of the present findings is that the programme content delivered by teachers through discussion, small group work and role plays, along with the animations as stimulus for reflection, was effective in improving students' ability to express and identify feelings. Emotion regulation is a significant skill contributing strongly to mental health and well‐being, but it is understood that children differ in their ability to identify and label internal emotional experience [42]. Emotional awareness develops through early and middle childhood [43] and while young children can report on their feelings and emotions, older children with improved language capacity and meta‐cognitive abilities are better able to reflect on their emotional experiences and in doing so regulate them [44].

Anxiety is a very common experience in children [45], and in this study and an evaluation was made of the effectiveness of the 6‐week intervention in reducing anxiety. Analysis of the ‘Fear of Negative Evaluation’ component showed no significant change from pretest to post‐test for the whole sample. Further analysis identified a group of children in the sample with low levels of FNE and the programme did not impact their level of anxiety. However, for a group of children who scored highly in terms of Fear of Negative Evaluation there was a significant decline in their anxiety across the intervention period. It is possible that this lessening of anxiety by this ‘vulnerable’ population is positively impacted by the intervention. It is now clear that the early identification of young people with social anxiety or social fears should be a concern to teachers [17]. Anxiety is one of the most frequently reported issues for young children and in the present study the focus was on the anxiety that arises out of peer relationships—social anxiety. Social anxiety often precedes the development of early onset major depression [46]. The early identification of young people with social anxiety or social fears should be a concern to educators. The findings from the present study suggested that the programme was most impactful for that subgroup of students who did report anxiety arising out of their everyday interactions with their peers. The findings suggested that the anxiety of being negatively evaluated by peers reduced across the intervention period and/or students learnt not to negatively evaluate others.

Schools have an accessible population of children that can be targeted for general, as well as specific, health promotion initiatives [17, 19, 47]. The focus on initiatives in schools has changed in accordance with World Health Organization [48] recommendations, towards a ‘settings’ approach, consistent with the concept of health promoting schools [49]. Our analysis suggested that teachers could effectively deliver and improve the mental health and well‐being of students, particularly, in relation to strengthening students understanding of emotions, emotional regulation and development of positive peer relations. This finding is important as student emotional awareness is required for their competent emotional functioning. Emotional awareness and emotion expression contribute to the developing in a child of their sense of subjective well‐being and capacity for adaptive resilience in the face of facing stressful situations [30].

### 5.1 Limitations of the Study

There are limitations in this pilot study which need to be acknowledged. The small number of participating schools and the school selection by of the year levels to participate limit the generalisability of the findings. The nested nature of the data needs to be noted along with the lack of a control or comparison group. A larger study that includes a bigger number and broader socio‐economic sample, with the inclusion of a control group or a wait listed group of schools. A larger sample size would enable a more robust examination of gender and year level differences and similarities with possible implications for developing more focussed intervention strategies. Ideally, some randomisation of students who complete the programme in each of the classes would occur. While schools and classrooms present important opportunities to implement mental health interventions there are particular challenges working in this environment which may limit the conduct of research, for example, the pressure on teachers to deliver on curriculum outcomes and the increasing complexity of student behaviour that teachers face. Miller et al. [23] have drawn attention to the innovations in study design presently being developed that take into account the challenges presented in schools to implementation science. As such the limitations identified in conducting this pilot study should be noted with consideration being given to new innovations in study design.

## 6 Summary

The current study evaluated an early intervention with young primary school students in the real context of the curriculum. A premise of the study is the need to provide further evidence‐based practice in mental health education and well‐being interventions. Our intervention used a six lesson multi‐component digitally delivered programme conducted by classroom teachers. Allowing for the limitations of the study the findings pointed to the suggestion that the programme was impactful in relation to promoting aspects of student well‐being, emotional development and in reducing elements of anxiety arising from fears regarding negative peer relations. Analysis pointed to associations between bullying and unhappiness at school, poor self‐esteem and self‐concept and a limited peer network. The findings are encouraging of further investigation with a larger more representative sample of students.

## Author Contributions

The research question, study design and methodology were developed by P.T.S. The intervention programme and measurement tool were developed by P.T.S. P.T.S collected the primary data, conducted all data analyses, interpreted the findings, prepared the tables and figures and wrote the manuscript with direction from S.P. and D.A. All authors reviewed the manuscript.

## Ethics Statement

All procedures performed in the studies involving human participants were in accordance with the ethical standards of the institutional and/or national research committee.

## Conflicts of Interest

The authors declare no conflicts of interest.

## Data Availability

The data that support the findings of this study are available from the corresponding author upon reasonable request.
